# Temporal evolution of arterial pulse pressure trajectories as early predictor of weaning success in venoarterial extracorporeal membrane oxygenation

**DOI:** 10.1177/02676591261439578

**Published:** 2026-03-30

**Authors:** Jeannine A. Hermens, Arno Fennema, Kim van der Tak, Daan Imholz, Libera Fresiello, Lara C. Pladet, Myrthe P. van Steenwijk, Christiaan L. Meuwese, Dirk W. Donker, Lex M. van Loon

**Affiliations:** 1 Department of Intensive Care Medicine, University Medical Centre Utrecht, Utrecht University, Utrecht, The Netherlands; 2 Cardiovascular and Respiratory Physiology, TechMed Centre, University of Twente, Enschede, The Netherlands; 3 Department of Intensive Care Adults and Cardiology, Thorax Center, Cardiovascular Institute, Erasmus Medical Center, Rotterdam, the Netherlands; 4 Department of Pediatric Intensive Care Medicine, Wilhelmina Children’s Hospital, Utrecht, The Netherlands

**Keywords:** venoarterial extracorporeal membrane oxygenation, cardiogenic shock, ECMO weaning, pulse pressure

## Abstract

**Introduction:** Weaning from venoarterial extracorporeal membrane oxygenation (VA ECMO) remains a major challenge because reliable indicators of cardiac recovery are lacking. Arterial pulse pressure (PP), a simple and continuously measured parameter, has been proposed as a surrogate of myocardial function and a potential predictor of weaning outcome.

**Methods:** In this retrospective study, 265 adult VA ECMO supported cardiogenic shock patients at a Dutch tertiary center were screened between 2011 and 2022, of whom 96 were eligible for analysis (mean age 56 ± 15 years, 32.3% female). Continuous, high-resolution hemodynamic data were analyzed over the first 36 h after ECMO initiation. Two dynamic PP metrics (absolute change and regression slope) were derived and compared between patients who were successfully weaned and those who were not. Associations were tested using mixed-effects models, logistic regression, while accounting for ECMO flow, vasoactive-inotropic score, and heart rate.

**Results: **PP was consistently higher from 12 h onward in successfully weaned patients (45.8%). Dynamic PP metrics showed significant associations with outcome (*p* < 0.001) and remained independent predictors after adjustment.

**Conclusion: **These findings suggest that an early rise in PP may aid to predict VA ECMO weaning success and supports its use as an accessible, real-time marker of myocardial recovery.

## Background

Venoarterial extracorporeal membrane oxygenation (VA ECMO) is increasingly used worldwide as a potentially life-saving intervention for patients with severe cardiogenic shock, serving as a bridge to myocardial recovery, durable ventricular assist device implantation, or heart transplantation^1,2^ While providing critical life support, prolonged VA ECMO carries risks of complications such as bleeding, thromboembolism, and infection, underscoring the importance of timely weaning.^3^ Conversely, premature discontinuation may precipitate relapse or worsening of heart failure, often necessitating emergent recannulation and compromising outcomes. Notably, a high mortality of up to 65% has been reported after initially successful decannulation,^4^ indicating the difficulty of reliably predicting durable weaning success.^5^ For these reasons, identifying an optimal weaning strategy and timing of decannulation is crucial.

Although multiple clinical parameters have been proposed to guide ECMO weaning, reliable and easy accessible real-time markers are still lacking, and a universally accepted standardized weaning protocol has yet to be established.^6,7^ Reported weaning prediction scores often rely on variables gathered before ECMO initiation,^8^ while commonly used prognostic parameters, such as clinical parameters, serum biomarkers and echocardiographic measurements, are subjective, time-consuming and costly, and perform inconsistently.^9–11^

Arterial pulse pressure (PP) is a continuously monitored and easily accessible bedside parameter closely related to cardiac contractility.^12^ Therefore, it carries great potential as an informative surrogate for cardiac recovery to support weaning considerations throughout the course of VA ECMO. Multiple studies have shown that an increase in PP is associated with successful VA ECMO weaning and reduced mortality, emphasizing its value as both a predictive and prognostic indicator.^4,13–19^ These studies, however, have primarily focused on static thresholds rather than a detailed temporal evolution of PP. Yet, PP is an inherently dynamic and multifactorial parameter, influenced not only by intrinsic myocardial contractility but also by ECMO blood flow, vasoactive and inotropic therapy, and heart rate.^20^ This underscores the importance of a thorough longitudinal assessment of individual VA ECMO courses to facilitate interpretation of PP within its clinical context. In this regard, the present study contributes by analyzing the temporal trajectory of PP in relation to physiological determinants that modulate it, and by evaluating its association with weaning outcomes in patients receiving VA ECMO support.

## Methods

### Study design and setting

A cohort study was conducted at the University Medical Centre Utrecht, the Netherlands, a tertiary referral center for advanced heart failure therapy. All adult patients receiving VA ECMO between 2011 and 2022 were screened for potential inclusion. The local ethics committee waived the requirement for informed consent due to the retrospective nature of the study (24U-1838).

### Patient selection and clinical management

Only patients with cardiogenic shock were included, with VA ECMO initiated in the presence of sustained hypotension or inadequate organ perfusion unresponsive to inotropes and vasopressors, and those with post-cardiotomy failure to wean from cardiopulmonary bypass. Patients were excluded if they received VA ECMO following lung transplantation or required additional mechanical left ventricular unloading, e.g. intra-aortic balloon pump or Impella microaxial pump. Patients were also excluded from analysis if they died from non-circulatory causes, i.e., post-anoxic brain injury, massive bleeding or refractory septic shock.

ECMO management followed institutional protocols, as described previously,^21^ initially focusing on restoring systemic perfusion, guided by mean arterial pressure (MAP), urine output, and lactate levels. During this stabilization phase, ECMO flow was typically maintained at full support (>4 L/min), aiming for a PP above 10 mmHg. After normalization of end-organ perfusion and lactate levels, ECMO flow was gradually reduced to 2 L/min in preparation for a weaning attempt, during which clinical optimization continued.^22^

The primary outcome was defined as successful weaning, which was established when patients survived after VA ECMO discontinuation through ICU discharge without requiring renewed mechanical circulatory support, ventricular assist device implantation, or heart transplantation within this timeframe.

### Data collection and preprocessing

Demographic, clinical and hemodynamic data were obtained from electronic health records (MetaVision; iMDsoft, Tel Aviv, Israel). Baseline characteristics included age, sex, body mass index, comorbidities, VA ECMO configuration, indication, and ICU mortality. Hemodynamic measurements, i.e., systolic and diastolic pressure (SBP, DBP), MAP, PP and heart rate, were recorded at one-minute intervals using an arterial line, typically placed in the right radial artery. VA ECMO parameters, including pump speed in revolutions per minute (rpm) and flow rate, were recorded hourly or at the time of manual adjustments. Pharmacologic support was quantified using the Vasoactive Inotropic Score (VIS), which was calculated on a minute-by-minute basis.^23^

To prepare the dataset for analysis, missing ECMO data were imputed using forward-filling methods until the next available value. Outlier values were removed using physiologic thresholds and a Hampel filter with a cutoff of three times the median absolute deviation.^24,25^ Based on visual inspection of signal trends, filter windows were set at 120 min for hemodynamic data and 5 hours for ECMO parameters. Missing values were linearly interpolated across time points after outlier removal. Patients were removed if baseline characteristics were unavailable, or if more than 25% of blood pressure data was missing, or if gaps of five or more consecutive hours of blood pressure data were present. All patients who passed quality control were included in the final analysis.

### Statistical analysis

Statistical analyses were performed using Python (version 3.13.2) and R (R Foundation for Statistical Computing, version 4.4.3). A two-sided significance level of 0.05 was applied. Continuous variables were assessed for normality by evaluation of histograms and reported as means with standard deviations or medians with interquartile ranges (IQR), depending on their distribution. Between-group comparisons were performed using the Student’s t-test for normally distributed data and the Wilcoxon rank-sum test for non-normally distributed data. Categorical variables were compared using Pearson’s chi-squared test.

To analyse temporal PP dynamics, a linear mixed-effects model with fixed effects for time (categorical), weaning outcome, and their interaction (and a random intercept per patient) was used to assess group differences in PP change relative to baseline (0 h). To facilitate statistical comparison at clinically meaningful evaluation points, the continuously recorded high-resolution data were tested at 12-h intervals after ECMO initiation.

Two additional dynamic PP metrics were calculated: the slope of the linear regression line over the first 36 h (PP_slope_) and the absolute change from hour 0 to hour 36 (ΔPP). The 36-h evaluation point was selected based on maximal effect size in the mixed-effects model and its clinical relevance as an early weaning assessment window.

Univariable logistic regression was used to examine associations between PP or other physiological parameters and weaning success. Variables significantly associated with outcome in univariable analysis were subsequently included in multivariable logistic regression to evaluate the independent association of PP while adjusting for potential confounders. Collinearity among predictors was assessed using pairwise correlations; variables with strong correlation (|r| > 0.7) were not entered jointly.

Physiological parameters potentially modulating PP (ECMO flow, RPM, VIS, heart rate, and MAP) were also assessed is relation to weaning outcome. Group-level temporal differences were evaluated to explore potential physiological relationships, and the similarity between their trajectories and PP was quantified using Spearman’s rank correlation coefficient.

## Results

### Study population

Of the 265 patients screened, 54 who received ECMO support following lung transplantation and 75 who received ventricular assist or unloading devices were excluded (Figure 1). An additional nine patients who died in the ICU from non-circulatory causes and 31 with insufficient data were also excluded. This left 96 patients for the final analysis, of whom 44 (45.8%) were successfully weaned from VA ECMO.

**Figure 1.**
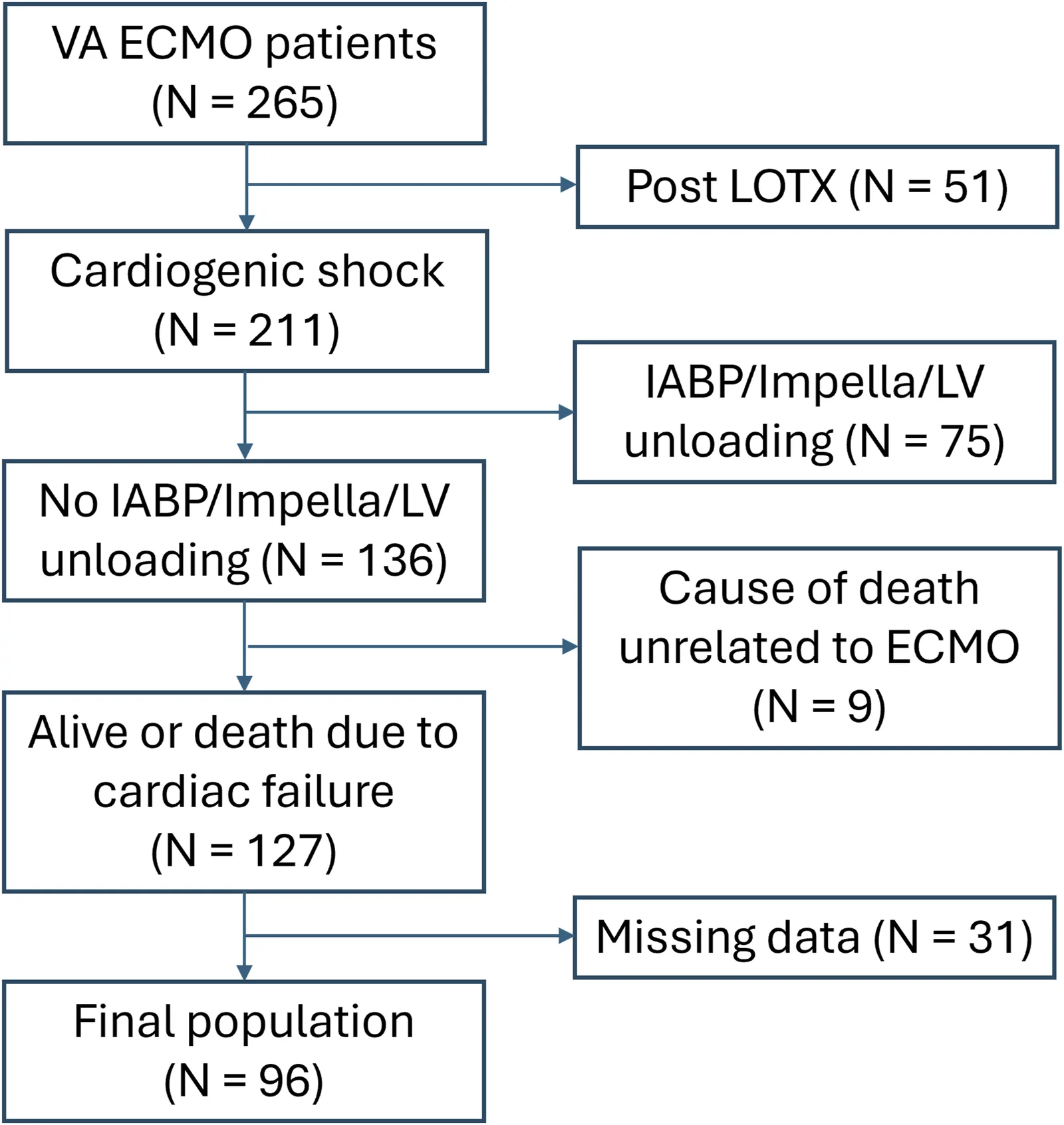
Patient inclusion and exclusion flowchart. Depicts the stepwise inclusion of patients from the initial VA ECMO cohort. LoTx: lung transplantation; IABP: intra-aortic balloon pump; LV: left ventricle.

### Baseline characteristics

No significant differences in baseline characteristics were observed between patients who were successfully weaned and those who were not (Table 1). The mean age was comparable between both groups. Patients in the weaning success group included a higher proportion of females and tended to have fewer cardiovascular comorbidities. The median duration of ECMO treatment was 4.1 days (IQR 2.4–6.1) in the weaning success group and 4.8 days (IQR 2.8–7.8) In both groups, the most common indication for VA ECMO was extended post-cardiotomy support (Table 1).

**Table 1.** Baseline characteristics of the study population stratified by weaning outcome.

	Weaning success (*N* = 44)	Weaning failure (*N* = 52)	*p*-value
Age (years)	55.0 ± 12.6	58.0 ± 16.1	0.242^a^
BMI (kg/m^2^)	28.0 ± 5.6	26.9 ± 5.3	0.321^a^
Sex female (%)	19 (43.2)	12 (23.1)	0.060^b^
Cardiovascular comorbidities n(%)	29 (65.9)	39 (75.0)	0.453^b^
Hypertension	17 (32.7)	11 (25.0)	
Diabetes	8 (15.4)	4 (9.1)	
Coronary artery disease	21 (40.4)	13 (29.5)	
CABG	10 (19.2)	4 (9.1)	
Chronic kidney disease	4 (7.7)	3 (6.8)	
Peripheral arterial disease	1 (1.9)	2 (4.5)	
Heart failure	16 (30.8)	16 (36.4)	
PCI	12 (23.1)	4 (9.1)	
CVA	6 (11.5)	5 (11.4)	
ECMO duration (days)	4.1 (2.4-6.1)	4.8 (2.8-7.8)	0.244^c^
ECLS indication n(%)			0.364^b^
*Post cardiotomy*	21 (47.7)	18 (34.6)	0.216^d^
Post-HTx/PGD	6 (13.6)	2 (3.8)	
*Ischemic cardiomyopathy*	4 (9.1)	12 (23.1)	0.099^d^
Myocardial infarction	3 (6.8)	8 (15.4)	
*ECPR*	7 (15.9)	6 (11.5)	0.564^d^
ECPR: Hypothermia	1 (2.3)	0 (0)	
ECPR: Refractory VT/VF	3 (6.8)	4 (7.7)	
ECPR: PEA/asystole	3 (6.8)	2 (3.8)	
*Non-ischemic cardiomyopathy*	12 (27.3)	16 (30.8)	0.823^d^
Myocarditis	2 (4.5)	1 (1.9)	
Congenital heart disease	0 (0)	1 (1.9)	
Pulmonary embolism	3 (6.8)	2 (3.8)	
Intoxication	0 (0)	1 (1.9)	
Other	4 (9.1)	2 (3.8)	
Cannulation site n(%)			0.300^b^
Peripheral	32 (72.73)	28 (54.85)	
Central	12 (27.27)	24 (46.15)	

Continuous variables are presented as mean ± standard deviation or median and interquartile range, as appropriate. Categorical variables are shown as counts (percentages).

CABG: coronary artery bypass grafting; PCI: percutaneous coronary intervention; CVA: cerebrovascular accident; HTx: heart transplantation; PGD: primary graft dysfunction; VT/VF: ventricular tachycardia/ventricular fibrillation; PEA: pulseless electrical activity. Statistical tests used are indicated with superscripts.

^a^Student’s t-test.

^b^Pearson’s chi-squared test.

^c^Wilcoxon rank-sum test.

^d^Fisher’s exact test.

### Pulse pressure

Table 2 and Figure 2 represent the mean trajectory of PP including 95% confidence intervals across the first week after ECMO initiation for both successfully and unsuccessfully weaned patients. A total of eight patients were excluded from the analysis because ECMO was discontinued within 36 h. From 12 h after the initiation of ECMO, patients who were ultimately successfully weaned exhibited a significantly higher PP. This difference was evident at all subsequent time points. The results from the linear mixed-effects model demonstrated a significant difference in PP trend over time between the weaning outcome groups with the largest effect size at 36 h. The mean PP_slope_ and ΔPP differed significantly between weaning success and failure groups with values of 0.42 ± 0.40 and 0.04 ± 0.31 mmHg/hour (*p* < 0.001), and 15.3 ± 14.3 and 1.5 ± 14.2 mmHg (*p* < 0.001), respectively (Figure 3).

**Table 2.** Temporal evolution of pulse pressure (PP) after ECMO initiation.

	Weaning success	Weaning failure	# patients	*p*-value^a^	Effect size	*p*-value^b^
0 h	29.2 (14.0–40.0)	18.5 (11.8–29.7)	[44, 52]	0.059		
12 h	31.1 (23.5–43.1)	18.7 (8.5–28.6)	[44, 52]	<0.001	6.44	0.016
24 h	39.1 (28.3–47.6)	18.4 (6.9–29.1)	[44, 52]	<0.001	12.34	<0.001
36 h	39.3 (33.5–49.1)	23.1 (9.2–31.1)	[42, 46]	<0.001	13.40	<0.001
48 h	42.3 (35.6–47.8)	24.0 (11.4–33.6)	[34, 44]	<0.001	12.25	<0.001
60 h	40.5 (33.8–48.2)	25.6 (7.8–38.2)	[33, 41]	<0.001	12.51	<0.001
72 h	37.4 (31.5–48.4)	25.1 (13.5–39.8)	[29, 37]	<0.001	10.50	<0.001

^a^Wilcoxon rank-sum test.

^b^Linear mixed model.

**Figure 2.**
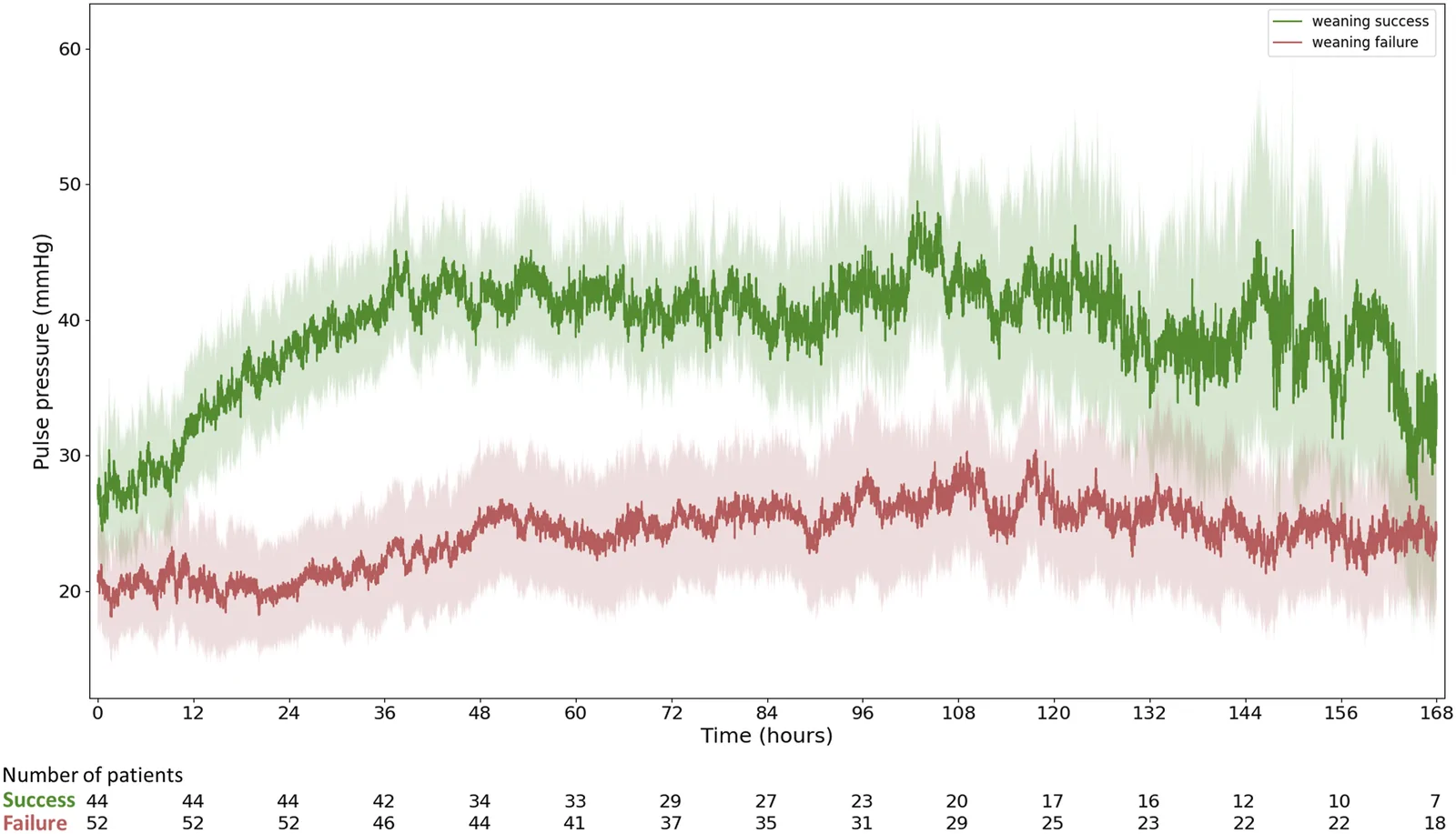
Group-averaged pulse pressure during the first week of ECMO support Mean (±95% CI) pulse pressure (PP) in patients who were successfully weaned (green) and those who were not (red). Below the graph, the patients at risk for the corresponding timepoint are given.

**Figure 3.**
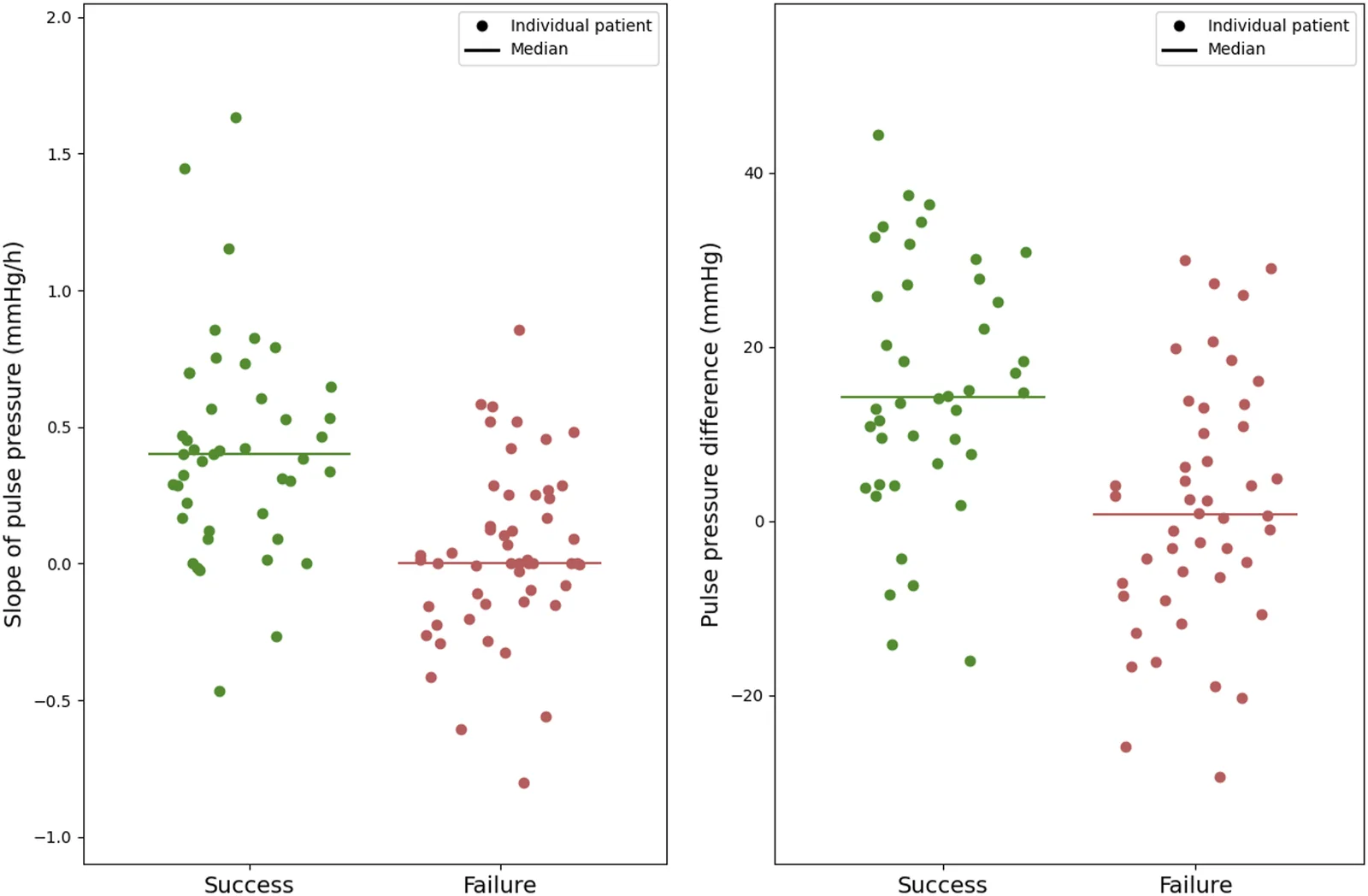
Early pulse pressure dynamics stratified by weaning outcome. Left: Distribution of regression slopes (PP_slope_) over the first 36 h. Right: Absolute change in pulse pressure (ΔPP) between 0 and 36 h. Colours denote weaning success (green) and failure (red).

ECMO rpm (after 24 h) and blood flow (from onset) differed significantly between groups, whereas VIS and heart rate did not (Figure 4). Furthermore, no difference in maintaining adequate MAP (>65 mmHg) during ECMO treatment was observed between both groups. Spearman’s correlations between PP and clinical parameters were generally weak and showed high inter-patient variability (Figure 5).

**Figure 4.**
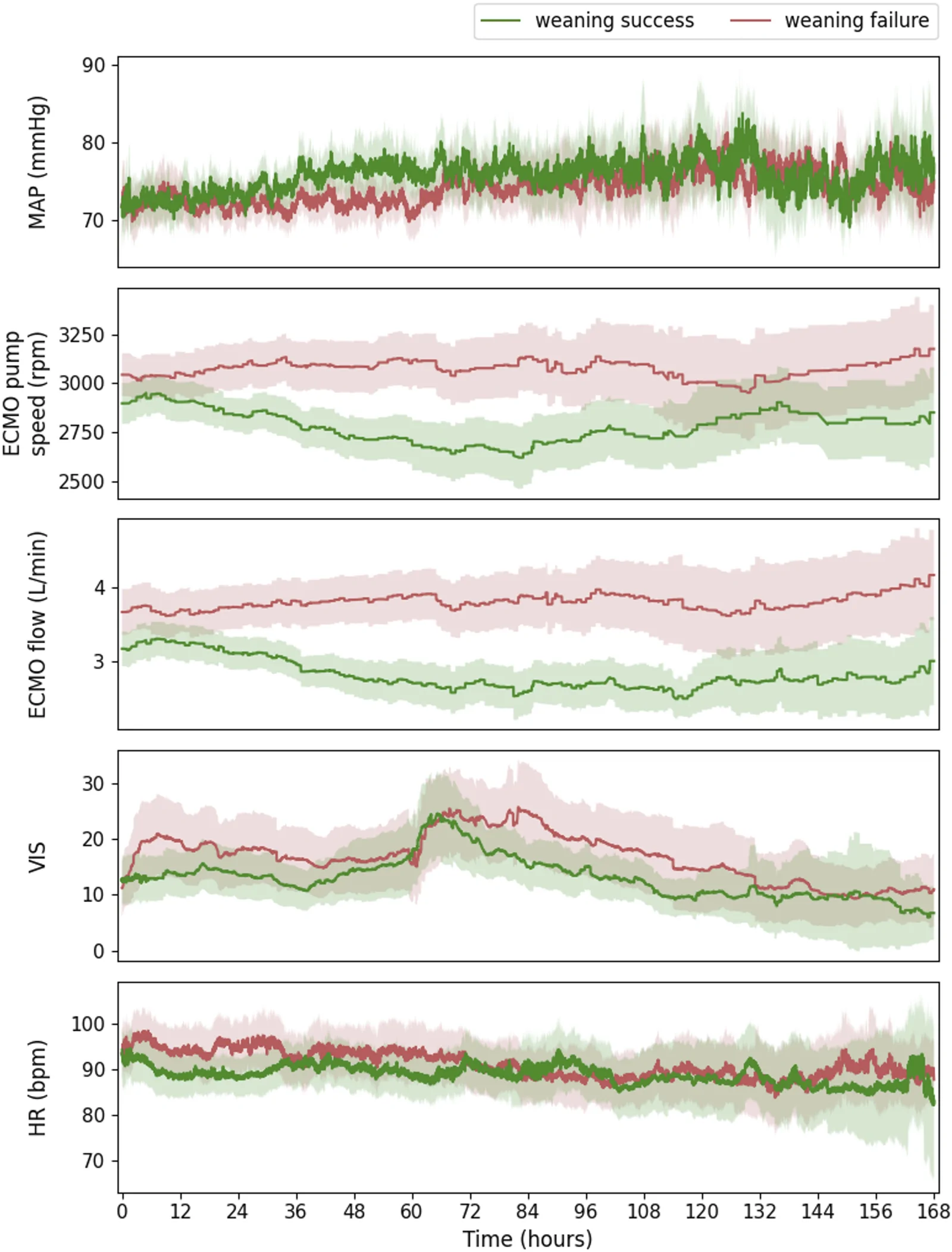
Hemodynamic profiles during ECMO support by outcome. Group-averaged trajectories of mean arterial pressure (MAP), ECMO pump speed (rpm), ECMO flow (L/min), vasoactive inotropic score (VIS), and heart rate (HR) during the first week after ECMO initiation for weaning success (green) and weaning failure (red).

**Figure 5.**
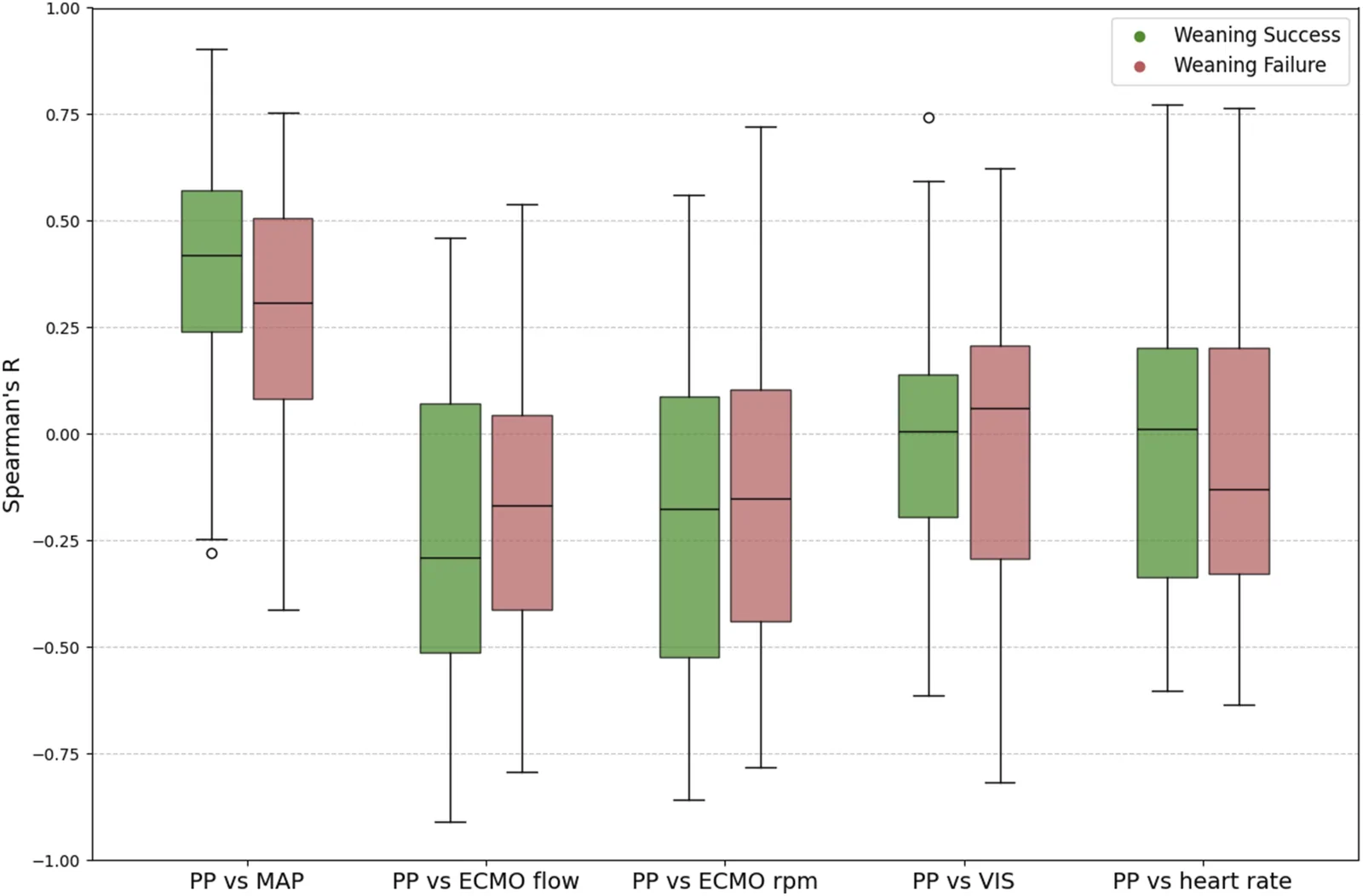
Patient-level correlations between pulse pressure and clinically related variables. Boxplots displaying the distribution of Spearman’s rank correlation coefficients between pulse pressure (PP) and mean arterial pressure (MAP), ECMO flow, pump speed (rpm), vasoactive inotropic score (VIS), and heart rate across patients from both groups.

Univariable logistic regression revealed a significant association of PP, PP_slope_ and ΔPP with weaning outcome (Table 3). Similarly, ECMO rpm and flow demonstrated a significant relationship with weaning outcome, whereas MAP, VIS, and heart rate did not. Multivariable logistic regression identified PP at 36 h as the only predictor significantly associated with weaning success when adjusted for ECMO flow and rpm, indicating that PP provides independent information beyond ECMO settings.

**Table 3.** Predictors of weaning in univariable and multivariable logistic regression.

	Univariable		Multivariable	
	OR [95% CI]	*p*-value	OR [95% CI]	*p*-value
PP	1.09 [1.05 1.14]	<0.001	1.08 [1.04, 1.14]	<0.001
PP_slope_	31.73 [6.17 163.24]	<0.001		
ΔPP	1.07 [1.03 1.11]	<0.001		
MAP	1.04 [0.99 1.1 ]	0.087		
ECMO rpm (×1000)	0.13 [0.04 0.45]	0.002	1.00 [1.00, 1.00]	0.425
ECMO flow	0.37 [0.22 0.64]	<0.001	0.89 [0.39, 1.98]	0.780
VIS	0.98 [0.96 1.01]	0.160		
Heart rate	1.00 [0.97 1.02]	0.706		

Odds ratios (OR) with 95% confidence intervals (CI) are reported. Multivariable models include pulse pressure (PP) and significant univariable covariates.

PP: pulse pressure at 36 h; PP_slope_,: regression slope of pulse pressure over the first 36 h; ΔPP: absolute change in pulse pressure between onset and 36 h; MAP: mean arterial pressure; rpm: ECMO pump speed (revolutions per minute); VIS: Vasoactive Inotropic Score.

## Discussion

In this cohort of VA ECMO-supported patients, a higher arterial PP, particularly an early increase within the first 36 h of support, was associated with successful weaning. Pulse pressure (PP) may thus represent an independent, physiologically meaningful marker of early cardiac recovery during VA ECMO, offering the great advantage of continuous bedside monitoring as readily available.

Current weaning trials rely strongly on cumbersome clinical, echocardiographic, and laboratory evaluations, often embedded in a multidisciplinary context. These approaches, while informative, are labour-intensive, time-consuming, and often subjective.^11^ In contrast, PP is derived from standard arterial blood pressure monitoring and offers continuous, real-time insights into the complex interplay between ECMO blood flow, native cardiac stroke volume and vascular compliance.^12^ As myocardial recovery leads to increased stroke volume, a rising PP may well indicate restoration of native cardiac function. This physiological concept is strongly supported by our findings, as successfully weaned patients showed a significantly higher PP early after ECMO initiation, with the steepest divergence within 36 h. While earlier studies support the correlation between higher PP values and favourable weaning outcomes,^4,13–19^ only a limited number examined PP evolution over time, often relying merely on infrequent or extrapolated measurements.^14,16^ Our high-resolution data extend prior findings by demonstrating not only that PP is higher in successfully weaned patients, but also that its increase occurs early after ECMO onset, suggesting a critical window to allow for discrimination between weanable and non-weanable patients at an early stage. These results not only reinforce the potential of PP, but more importantly, highlight that its dynamic trend, rather than a single absolute value, offers additional clinical insights into early cardiac recovery under VA ECMO support. However, while current findings suggest a potential association between PP and cardiac recovery, this observation should be interpreted as hypothesis-generating awaiting validation in larger populations and prospective studies.

PP is determined by multiple physiological factors besides cardiac contractility, such as vascular tone and cardiocirculatory loading conditions. Therefore, it can thus be affected by alterations in ECMO blood flow, vasoactive therapy, arterial compliance, and heart rate.^20^ Although these factors are theoretically expected to influence PP, our findings do not identify them as primary determinants of the observed divergence in PP trajectories between outcome groups. Although arterial stiffness may influence PP independently of stroke volume,^12^ its variation over the 36-h timeframe is negligible and therefore unlikely to have impacted the observed PP trends. Still, inter-individual differences in arterial compliance, related to age or vascular risk, may influence PP behavior and deserve further investigation to potentially stratify weanability. Future studies should explore whether adjusting PP for baseline vascular properties enhances its predictive value.

PP is also modulated by inotropes and vasopressors, and adjustment for the VIS has previously been shown to improve its predictive validity.^13^ In our cohort, however, VIS did not differ between outcome groups, suggesting that it may not adequately capture myocardial and vascular responsiveness. Notably, inotropic requirements are physiologically determined by the patient’s recruitable contractile reserve and may dynamically change over time. Yet, while aiming for adequate systemic perfusion and PP under VA ECMO, meticulous tailoring of inotropic support in relation to the individual’s myocardial contractile reserve is virtually impossible in daily clinical practice. Consequently, a given inotropic dose may exert very heterogeneous effects on the native cardiac contractility and, in turn, on PP across patients.

Furthermore, our routine practice is to maintain extracorporeal flow at the lowest possible, clinically acceptable level, typically around 2 L/min, while deploying active gradual tapering of ECMO blood flow shortly after circulatory stabilization.^22^ As PP showed no consistent correlation with MAP, ECMO parameters, or heart rate, it supports the interpretation that its rise reflects native cardiac recovery. Notably, multivariable analysis confirmed PP at 36 h as the only variable independently associated with weaning success.

Given the above findings, the divergent PP trajectories observed as early as 12 h after ECMO initiation appear to reasonably reflect improvement in native cardiac contractility. Therefore, evaluating PP trends, rather than the interpretation of isolated timepoints, has great potential as a robust and physiologically grounded indicator of myocardial recovery in cardiogenic shock patients during VA ECMO support.

### Limitations

The dynamics of PP may depend on the underlying cause of cardiogenic shock. In our cohort, comprising a substantial number of post-cardiotomy patients, rapid PP improvement was common, consistent with recovery from transient postoperative myocardial stunning.^26^ In contrast, conditions such as acute myocardial infarction, myocarditis or chronic dilating cardiomyopathies may show delayed, less outspoken or even lack of significant improvement in PP trajectories,^27^ limiting the extrapolation of PP as a predictor of weaning success in individual cases.

Furthermore, patient inclusion spanned 11 years, during which practice patterns evolved, notably with a shift from central to peripheral cannulation. Although both configurations pressurize the aortic compartment, the relative contributions of antegrade and retrograde flow kinetics to radial PP remain complex, difficult to quantify, and incompletely understood.^28^ Also, the more frequent use of central cannulation in the early years may have been associated with higher complication rates, such as mediastinal bleeding and infection during VA ECMO support, potentially reducing the likelihood of successful weaning.

The retrospective design of this study introduces inherent risks of bias, and not all physiological parameters potentially modulating PP (*e.g.,* fluid balance, ventilator settings) could be accounted for. The definition of weaning success, although consistent with current practice, may differ from other studies, potentially affecting generalizability.

### Future directions

Development of standardized weaning protocols may benefit from incorporating PP trends alongside established clinical indicators. Computational physiological models offer promising opportunities to understand PP behaviour by simulating cardiovascular dynamics under VA ECMO, including ventriculo-arterial coupling, heart–lung interactions, and relevant modulatory parameters.^29–31^ These reduced-order models can support mechanistic interpretation of patient-specific PP trajectories and may ultimately strengthen clinical decision-making.^32^

Integrating such models into real-time decision support tools could enable automated correction of PP for key influencing factor, such as vascular tone, ECMO settings, and pharmacologic support, moving toward a truly individualized, continuously monitored weaning strategy.^9^ Beyond passive monitoring, model-driven perturbation protocols (e.g., stepwise reductions in ECMO flow or inotropes) could serve as dynamic challenges to test patient-specific contractile myocardial reserve through PP responses in a quasi-continuous way. This personalized, physiology-informed approach may reduce reliance on resource-intensive assessments and enhance safe, efficient weaning.

## Conclusion

In VA ECMO–supported patients with cardiogenic shock or post-cardiotomy failure, an early rise in arterial PP is a promising predictor of weaning success. As a continuously available bedside monitoring parameter, PP offers physiologically meaningful and easily accessible insights into the individual patient’s cardiac recovery. Our findings support the integration of PP trends, rather than static thresholds, into standardized weaning protocols and advanced future clinical decision-making tools, with a great potential to improve both efficiency and consistency of weaning assessments.

## Appendix

### List of abbreviations

AUC: Area under the curve
CI: Confidence interval
DBP: Diastolic blood pressure
IABP: Intra-aortic balloon pump
IQR: Inter-quartile range
MAP: Mean arterial pressure
OR: Odds ratio
PP: Pulse pressure
ROC: Receiver operating characteristic
rpm: Revolutions per minute
SVR: Systemic vascular resistance
SBP: Systolic blood pressure
VIS: Vasoactive inotropic score
VA ECMO: Venoarterial extracorporeal membrane oxygenation
